# Integrative approaches to nutrient management in tomato cultivation for improved sustainability and productivity

**DOI:** 10.3389/fpls.2025.1626136

**Published:** 2025-09-24

**Authors:** Yubo Li, Ruifang Zhang, Chi Zhang, Qingyun Li, Lanchun Nie, Can Wang, Xin-Xin Wang

**Affiliations:** ^1^ College of Horticulture, Hebei Agricultural University, Baoding, China; ^2^ State Key Laboratory of North China Crop Improvement and Regulation, Hebei Agricultural University, Baoding, Hebei, China; ^3^ College of Resources and Environment, Hebei Agricultural University, Baoding, China; ^4^ College of Land and Resources, Hebei Agricultural University, Baoding, China; ^5^ Spices and Beverage Research Institute, Chinese Academy of Tropical Agriculture Science, Wanning, Hainan, China

**Keywords:** tomato, soil fertility, nutrient management, integrated systems, yield and quality

## Abstract

Tomato is a vital crop within agricultural production systems and ranks among the most in-demand vegetables on the market, but tomato production faces significant challenges due to long-term cultivation practices, including soil successive cropping obstacles, nutrient imbalances, reduced microbial diversity, and the accumulation of allelopathic substances. Previous studies show that tomatoes exhibit substantial differences in yield and quality between integrated and conventional systems, primarily attributed to its high nutrient demands. This review synthesizes the most relevant scientific literature worldwide to examine the current state of knowledge regarding crop nutrition and soil fertility management in tomato production systems. It systematically analyzes the impacts of nutrient solutions, green manures, soil amendments, and biostimulants on both tomato yield and quality. The main findings indicate that conventional management methods lead to constrained tomato yields due to degraded soil fertility and inadequate nutrient supply. Therefore, integrated soil-tomato system strategies are required to enhance productivity and meet consumer demands. Additionally, this review uniquely integrates multidisciplinary approaches to highlight synergistic strategies for optimizing both yield and quality. We identify a critical gap in long-term comparative studies on soil-tomato system management and emphasize the need for consumer-oriented quality metrics in future research. By synthesizing global evidences, this work provides a comprehensive framework for sustainable tomato production beyond conventional nutrient-focused practices.

## 1 Introduction

Tomato (*Solanum lycopersicum* L.) is an extensively cultivated vegetable to meet the dietary needs of populations worldwide, as its enriched with vitamin C, antioxidants, and lycopene. In the context of ongoing advancements in economic conditions and living standards, it is essential to prioritize the exploration of taste and nutritional qualities alongside agricultural yield, particularly in crops such as tomatoes (Perveen et al., 2015; Kumar et al., 2022). Breeders and researchers are dedicated to developing tomato varieties that exhibit superior flavor and quality, with the goal of meeting market demands and enhancing the overall economic efficiency of the tomato industry (Klee, 2010; D’Amico et al., 2024).

Soil nutrient dynamics play a pivotal role in tomato productivity and fruit quality (Masih et al., 2020; Sharma et al., 2023). Soil fertility management is vital for a optimized nutrient level and plant development such as optimal pH, electrical conductivity (EC), and nitrogen levels showed positive effects on plant height, length, and width in tomatoes (Putranta et al., 2019). However, conventional intensive farming, particularly in greenhouse systems, often relies on excessive synthetic fertilizers, leading to soil acidification, nutrient imbalances, and secondary salinization (Liu et al., 2021). These issues not only hinder plant growth but also threaten the sustainability of agricultural systems. Organic fertilizers enhance soil quality, stability, and microbial diversity by altering soil microbial composition (Zhou et al., 2024). Excessive or improper use can promote surface water eutrophication and chemical or biological pollution, ultimately reducing soil fertility and adversely impacting vegetable yield and quality over time. Therefore, the judicious application of organic fertilizers and biostimulants is crucial for improving soil health and promoting the sustainable development of facility agriculture (Thi Kieu Oanh et al., 2023; Jana et al., 2024).

**Figure 1:**
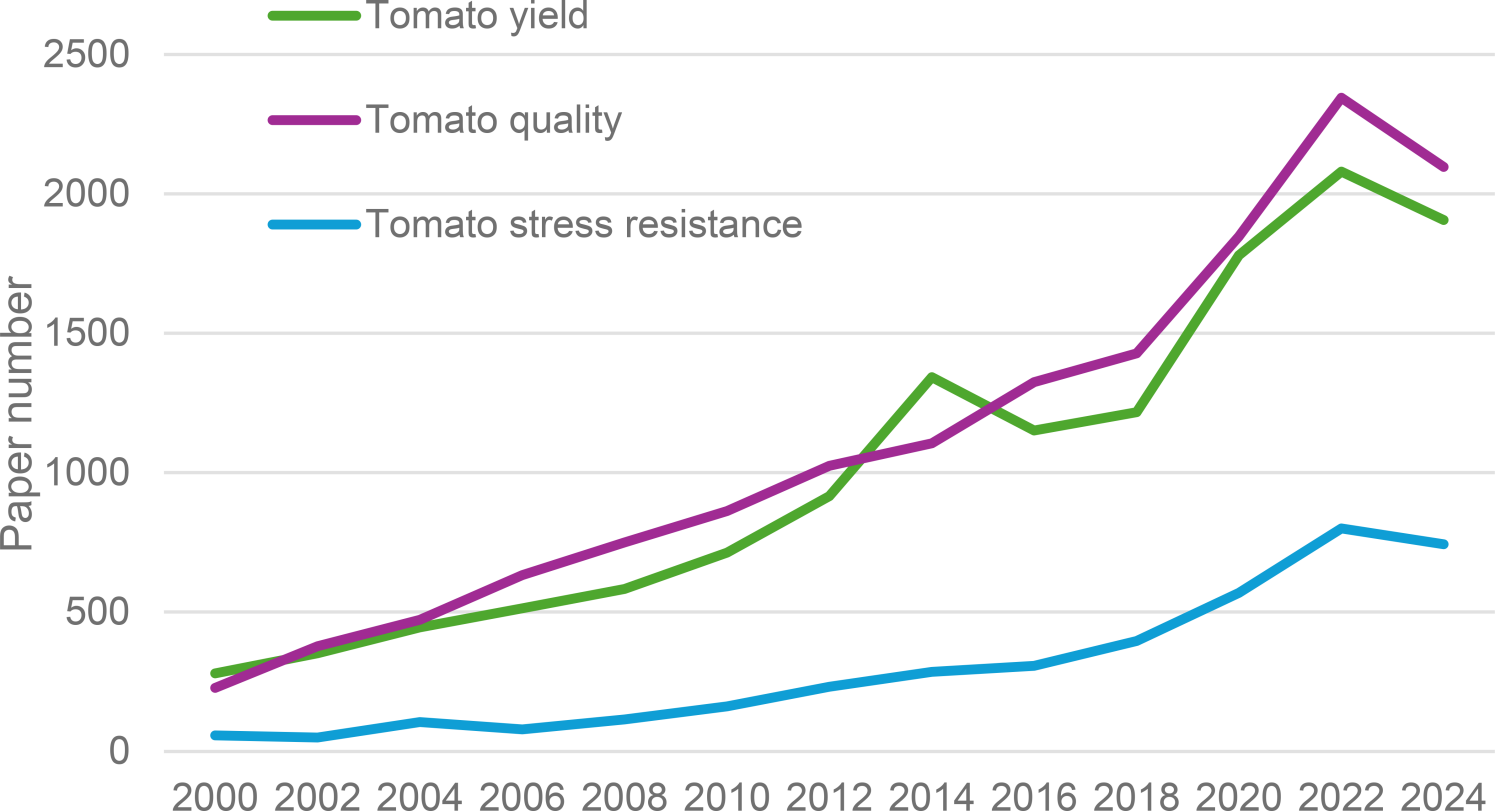
Annual counts of journal articles indexed in the Web of Science database from 2000 to 2024 containing the keywords “tomato yield,” “tomato quality,” and “tomato stress resistance.

Recent advances in soil fertility research underscore the potential of integrated nutrient management (INM) to reconcile yield and quality objectives in tomato production, with bibliometric analysis indicating a threefold increase in relevant studies since 2000 (**Figure 1**). However, unregulated organic inputs may contribute to nutrient leaching and eutrophication, necessitating precision management strategies. Despite this growing research focus, critical knowledge gaps remain concerning the trade-offs between short-term productivity and long-term soil health, the complex interactions between organic amendments and microbial consortia, and the practical scalability of precision nutrient delivery systems for smallholder farmers. These findings are particularly relevant for transitioning from conventional to integrated production systems, where the synergy between nutrient management and soil health can lead to more resilient and economically viable tomato cultivation. This review therefore synthesizes the latest of research to evaluate the efficacy of various soil fertility management strategies including optimized fertilization, biostimulants, and soil amendments in enhancing both tomato yield and quality, while critically assessing their impacts on fruit physicochemical properties, nutritional profiles, and economic viability to identify key priorities for sustainable intensification.

## 2 Effects of nutrient solution on tomato production

### 2.1 The effects of nutrient solution application on soil-grown tomatoes

**Figure 2:**
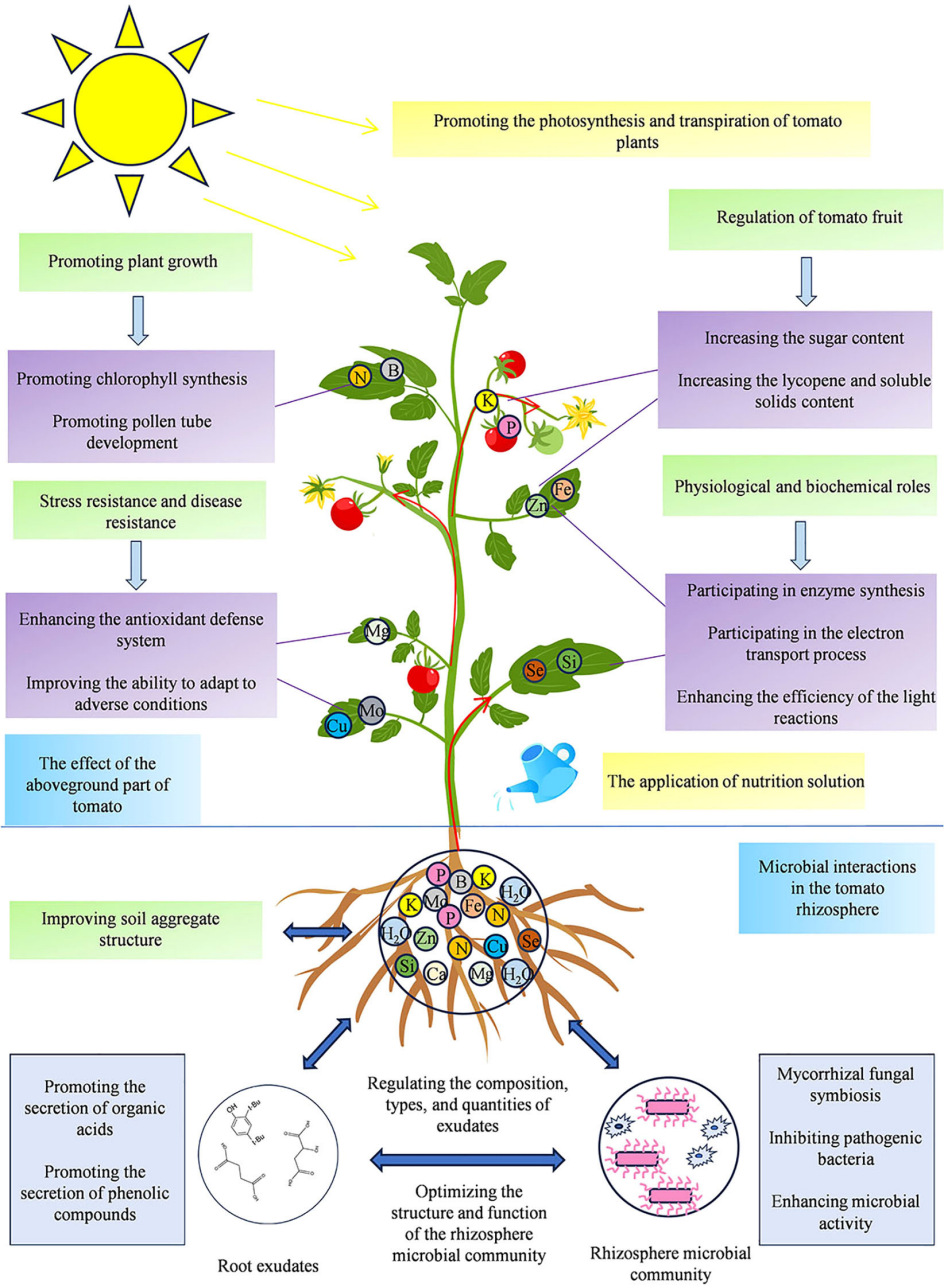
A systematic outline of nutrient solution utilization into plant and its effects on the yield, growth, defense, physiological and chemical mechanism in tomato plants.

Optimized nutrient solutions (ONS) markedly enhance fertilizer efficiency and tomato fruit quality (**Figure 2**). Studies show that adjusting EC and organic components (e.g., ONS) can increase total soluble solids (TSS) by 0.7%, soluble sugars by 23.3%, and organic acids by 33.4%, directly improving flavor and marketability (Ma et al., 2021; Lu et al., 2022). Additionally, optimal drainage rates with elevated EC promote sugar and aromatic compound accumulation (Ou et al., 2023).

However, imbalanced or excessive nutrient solutions may counter these benefits. High nitrogen/potassium concentrations can cause leaf chlorosis, fruit cracking, and yield loss (Gholamnejad et al., 2023; Xie et al., 2024), while prolonged over-application risks soil salinization and root dysfunction (Tarolli et al., 2024). Notably, the same EC levels that enhance sugar accumulation at optimal ranges may induce salt stress if exceeded, highlighting the need for precise management.

Likewise, the implementation of ONS in commercial production systems faces multiple challenges. Over reliance on nutrient solutions often leads to over fertilization especially under non precision based management (Fathidarehnijeh et al., 2024). This problem is exacerbated in systems without real time monitoring where imbalances in electrical conductivity or pH may accumulate resulting in nutrient leaching, soil salinization, and reduced microbial diversity (Tarolli et al., 2024; Mohmed et al., 2025). Furthermore economic and technical barriers such as the high cost of sensor based systems and the need for skilled labour limit the scalability of ONS in smallholder and resource limited settings (Awal et al., 2025). These limitations emphasise the necessity of integrating ONS with other sustainable practices like organic amendments and biostimulants to enhance system resilience and reduce environmental impacts.

Therefore, achieving high-quality yields requires a trade-off between nutrient optimization and salt stress mitigation, including dynamic adjustments of EC, pH, and drainage rates based on real-time plant responses (Putranta et al., 2019; Langenfeld et al., 2022). While nutrient solution optimization demonstrates significant potential for enhancing tomato quality, its long-term sustainability remains uncertain. Current research predominantly focuses on short-term agronomic effects, leaving critical gaps in our understanding of how continuous nutrient solution application impacts soil health over extended periods. Additionally, the economic viability of organic versus inorganic nutrient solutions in commercial-scale production systems requires rigorous assessment, particularly in resource-limited settings. Further complexities arise when considering climatic variability, as optimal nutrient management strategies must be adapted to seasonal conditions, such as summer’s high temperatures versus winter’s reduced light availability to maintain consistent yield and quality. To address these challenges, future studies should leverage advanced multi-omics methodologies, including metabolomics and microbiome analysis, to holistically optimize nutrient formulations. Such approaches could simultaneously maximize crop performance while reducing adverse environmental impacts, ensuring a balance between productivity and ecological stewardship.

### 2.2 The effects of nutrient solution application on hydroponically grown tomatoes

Modern agriculture has widely used hydroponics as an efficient soilless method for tomato production (Al-Gaadi et al., 2024). Tomato plants grown hydroponically depend on the formulation and maintenance of the nutrient solution, which has a direct impact on yield and quality (Hochmuth and Hochmuth, 2018). An adequate availability of nutrient solutions is essential to enhance the plant growth and development at all growth stages to maintain the of tomatoes, equilibrium between vegetative and reproductive stages (Liu et al., 2024). High-quality hydroponic items are becoming more and more in demand, and buyers are willing to pay more for hydroponic tomatoes (D’Amico et al., 2024). However, the economic viability of hydroponic systems remains questionable for small-scale farmers due to high initial infrastructure costs and energy demands for lighting and climate control. The premium prices hydroponic tomatoes command may not offset these expenses unless production is scaled significantly, raising concerns about accessibility and equity in agricultural innovation.

Tomato plants can be successfully grown using hydroponic tanks with the necessary modifications in a variety of environments, such as indoors and greenhouses. In comparison to applying the same fertilizer every two weeks and not replenishing the nutrient solution, renewing the nutrient solution every two weeks enhanced the leaf area and fresh weight of tomato plants by 18% and the 28%, respectively (Solis-Toapanta et al., 2020). The closed hydroponic system offered significant advantages in terms of water and fertilizer conservation, allowing nutrient solution consumption by 96% and fertilizer consumption by 97% without adversely affecting crop yield provide substantial benefits regarding water conservation and fertilizers (Fayezizadeh et al., 2021). Desalinated seawater (DSW) used in hydroponic systems instead of conventional water resources is most accurate alternative tofacilitating nearly year-round continuous production and elevated crop yields. Irrigation with DSW sepreate and along with conventional water sources did not impact tomato quality (Antolinos et al., 2020). However, desalination is an energy-intensive process that contributes to carbon emissions unless powered by renewable energy. Relying on DSW may simply shift water scarcity challenges from freshwater sources to energy demands, without addressing the root causes of resource depletion. A single cherry tomato plant could produce up to 682 g when grown hydroponically using a deep bed system (DBS) and irrigated with purified agricultural wastewater. This shows how agricultural waste can be used and provides a sustainable method of recycling agricultural wastewater (Afonso et al., 2023). Using DSW and agricultural wastewater to grow tomatoes hydroponically is a new way to recycle agriculture that effectively uses marine resources while reducing need on traditional freshwater sources. While wastewater recycling is commendable, potential contamination risks from heavy metals or pathogens must be rigorously managed. Without strict regulatory oversight, the use of treated wastewater in hydroponics could introduce food safety hazards, undermining consumer trust in soilless agriculture. The substantial upfront investment required for hydroponic systems makes their economic viability heavily contingent on high-value crops whose market prices can fully offset costs and generate surplus, thereby restricting their adoption for lower-margin produce (Souza et al., 2019). In addation, the nutrient uptake process in hydroponic systems critically affects crop yield and quality, influenced by nutrient interactions, availability, and chemical forms in the growth medium (Valentinuzzi et al., 2015). While hydroponic systems demonstrate superior operational cost-efficiency compared to conventional soil-based agriculture post-establishment, they present distinct technical limitations. The primary challenges include non-uniform nutrient distribution throughout the solution and heightened vulnerability to waterborne pathogen proliferation (Suárez-Cáceres et al., 2021; Sangeetha and Periyathambi, 2024). These constraints necessitate rigorous implementation of advanced crop health surveillance protocols and precision management strategies by cultivators. The focus should remain on holistic sustainability rather than isolated technological fixes.

## 3 The effects of green manure on tomatoes

Green manure is a crucial type of organic fertilizer derived from green plant materials used to improve soil structure, soil fertility, promotes nutrient availability and increases agricultural productivity (Wang et al., 2025). This agricultural practice involves cultivating specific green manure crops, collecting wild green manure species which are then incorporated into the soil through plowing or composting (Behera et al., 2025; Kama et al., 2025). While studies demonstrate benefits such as enhanced tomato yield, nutrient uptake, and soil quality, these findings may not be universally applicable due to contextual factors like soil types, climate, and management practices.

For instance, incorporating leguminous green manure is helpful to increase tomato fruit yield by 10%-30% relative to animal manure alone which directly supports tomato growth (Gatsios et al., 2019). However, this advantage varies across agroecological conditions, and improper incorporation timing or excessive use may disrupt soil balance or compete with cash crops for resources. Additionally, green manure significantly increased soil microbial biomass carbon (MBC) and microbial biomass nitrogen (MBN) by 20.0% and 18.5%, respectively (Behera et al., 2025). Nevertheless, such improvements may come with trade-offs, such as short-term nitrogen immobilization or pathogen risks under certain green manure regimes.

Green manure demonstrates substantial potential in tomato production systems (**Table 1**). This agricultural practice enhances soil fertility and structural integrity by providing essential macronutrients for optimizing tomato growth parameters and yield potential, including nitrogen, phosphorus, and potassium (Wei et al., 2025). However, claims of universal improvements in nutrient cycling and plant vigor require further scrutiny, as the effectiveness of green manure depends on decomposition rates, microbial communities, and farming practices, factors often overlooked in short-term studies. Furthermore, while green manure is often promoted for its carbon sequestration potential, long-term stability depends on complex interactions that are rarely examined in depth (Behera et al., 2025).

**Table 1:** The benefits of green manure for tomato production in different countries.

Green manure crops	Country	Planting/application pattern	Main benefits	References	
Oats (*Avena sativa* L.) and Barley mixture (*Hordeum vulgare* L.); Rye (*Secale cereale* L.); Brown Mustard (*Brassica juncea* L.); Flax (*Linum usitatissimum* L.); Pigeon Bean (*Vicia faba* L. var. *minor*)	Italy	Monoculture	Enhanced nitrogen availability; Increased marketable tomato yield; Improved nitrogen uptake by tomato; Reduced need for external fertilizers; Tomato quality maintenance	(Lenzi et al., 2009)	
Jack Bean (*Canavalia ensiformis*); Velvet Bean (*Mucuna pruriens*)	Ghana	Monoculture and intercropping	Reduced cash expenditure on fertilizer; Reduced weed growth; Possible benefit to subsequent crops; Lower rates of abortion and flower drop (due to lower temperatures)	(Dorward et al., 2003)	
Vetch (*Vicia villosa* Roth.); Barley (*Hordeum vulgare* L.)	Italy	Monoculture and intercropping	Reduced nitrate leaching; Enhanced biomass accumulation; Improved leaf area index (LAI); Higher yield potential	(Farneselli et al., 2018, 2020)	
Faba bean (*Vicia faba* L.); Alfalfa (*Medicago sativa* L.)	Greece	Mobile green manure	Increased soil nitrogen availability; Sustainable nitrogen input through biological nitrogen fixation (BNF); Higher economic returns due to increased yield	(Gatsios et al., 2021)	
Jack bean (*Canavalia ensiformis* DC); Sun hemp (*Crotalaria juncea* L.); Dwarf velvet bean (*Mucuna deeringiana* (Bort)); Mung bean (*Vigna radiata* (L.) Wilczek); White lupine (*Lupinus albus* L.); Cowpea bean (*Vigna unguiculata* (L.) Walp)	Brazil	Intercropping	Increased N transfer to cherry tomato; Higher N concentration in leaves and fruits; N transfer increases with tomato development; Sufficient N supply for cherry tomato	(Salgado et al., 2021)
Mexican sunflower (*Tithonia diversifolia*); Banana (*Musa* spp) leaves	Nigeria	Individual or combined application	Improved soil physical properties; Enhanced soil chemical properties; Increased tomato growth and yield; Enhanced soil mineral contents; Cost-effective and sustainable	(Adekiya, 2019)
Soybean (*Glycine max* L. Merr.); Indigofera (*Indigofera tinctoria* L.); Mungbean (*Vigna radiata* L. Wilcz.)	China and Philippines	Monoculture	Increased tomato yield; Enhanced nitrogen uptake; Improved soil fertility; Reduced need for synthetic fertilizers; Sustainable soil health;	(Tho Nnissen et al., 2000)

Beyond agronomic benefits, practical application remain understudied. Green manure species selection, frequently presented as straightforward, is highly sensitive to local conditions such as rainfall, soil pH, and microbial activity. Moreover, economic and labor constraints, including land opportunity costs and mechanization limitations for smallholder farmers, are frequently neglected in the study despite their critical influence on adoption rates. A more nuanced assessment is needed to determine the feasibility and effectiveness of green manure across diverse agricultural systems.

## 4 Biostimulants in tomato cultivation

### 4.1 Humic acid

Humic acids are natural organic substances found in soil as a result of the chemical breakdown and decomposition of plant matter, animal waste, and microorganisms due to microbial activity (Hayes, 1983; Calvo et al., 2014). By triggering biochemical and metabolic processes within plant cells and either directly or indirectly boosting mineral nutrition, humic acids can have biostimulant effects on plants, promoting growth (Shah et al., 2018; Quijia Pillajo et al., 2024; Zamljen et al., 2024). Moreover, humic acids particularly influence the growth hormones to facilitate the lateral and primary root development and regulate the metabolism of the root system (Zandonadi et al., 2007). These compounds stimulate the activity of plasma membrane H^+^-ATPase in roots, threby boosting the proton gradient in the cell membrane (Zandonadi et al., 2010; Jindo et al., 2012). This stimulation facilitate the nutrient absorption and concurrently influencethe expression of relevant genes (Zandonadi et al., 2010; Jindo et al., 2012). Addationally, humic acid improve soil structure and nutrient availability and strengthen the plant resilience to environmental stressors (Maffia et al., 2025).

Humic acid treatment resulted in a 1.5- to 2.6-fold increase in the number of lateral roots in tomato plants. Conversely, lateral root length exhibited an even more pronounced enhancement, ranging from 4.05- to 22.8-fold (Dobbss et al., 2007). This phenomenon was attributed to the similarity between the effects of humic acid and the stimulatory responses induced by the application of exogenous growth hormones regardless of their concentrations whether applied in small or large quantities (Cordeiro et al., 2011; Rathor et al., 2024). Application of 120 L/ha humic acid considerably enhanced soluble solids content, titratable acidity, tomato plantheight, stem diameter, SPAD, and yield (Asri, 2021). Humic acid also enhance the plant tolerance in response to osmotic stress by modulating the phytohormone and antioxidant metabolism, which promotes plant development and interestingly influences the modified the composition of the inter-root endophytic bacterial community (Lengrand et al., 2024). Pre-treatment with 4 mM humic acids significantly increased H^+^-ATPase activity by 60% and maintain the maximum quantum yield of Photosystem II (PSII; Fv/Fm) and significantly reduce the lipid peroxidation levels. These combined effects maintain plant growth parameters and substantially reduce salt-induced oxidative damage in tomato plants (Souza et al., 2021). However, excessive use of humic acid may cause tomato infection by root rot bacteria and elevate the prevalence of tomato root rot (Yigit and Dikilitas, 2008).

Although extensive research has substantiated the efficacy of humic acid applications in tomato cultivation, several criticallimitations warrant further investigation. Firstly, existing studies has predominantly focused on assessing short-term growth parameters and yield metrics, resulting in a limited understanding of the long-term effects of humic acids on sustainable tomato cultivation practices and their subsequent impacts on soil ecosystem dynamics. Secondly, variations in the source, extraction technique, and application method of humic acid across different studies complicate the comparison of experimental results. Thirdly, additional research is essential to determine the optimal dosage and frequency of humic acid treatments across various soil types and climatic conditionsSuch research is crucial to optimize application protocols and establishing consistency and adaptability across various agricultural environments and management practices.

### 4.2 Arbuscular mycorrhizal fungi

Arbuscular mycorrhizal fungi (AMF) are soil microorganisms, considered as plant root symbionts globally that establish a symbiotic association with plant roots (Smith et al., 2003). Most vegetable crops has potential to act as host plants for AMF including tomato, which can enhance nutrition and water availability, promote tolerance to environmental stressors, root and nematodes diseases (Smith et al., 2003; Baum et al., 2015; Flor-Peregrín et al., 2016; Leventis et al., 2021). However, the extent of these benefits may vary depending on environmental conditions, AMF species, and host genotypes. For instance, Yu et al. (2022) observed a 20% increase in root length and 15% improvement in root surface area in AMF-inoculated tomatoes, but similar studies in different soil types or climates might yield divergent results. Consequently, the optimized implementation of AMF to enhance yield and quality is essential for advancing the sustainable growth of the tomato-producing sector.

The synergistic interaction between AMF and plant growth-promoting bacteria (PGPB) demonstrates considerable potentialto attain sustainable agriculture. The synergistic application of AMF along with required fertilizer helps to improve the tomato growth and 13% yield compared to the non-AMF-inoculated plants, although a 50% reduction in chemical fertilizer was implemented (Ziane et al., 2017; Bernados et al., 2024). However, the mechanisms by which AMF enhances phosphorus uptake or alters root exudates remain unclear and require further mechanistic investigation.

Utkhede (2006) reported a 46% reduction in root rot and 15% yield improvement, the efficacy of AMF against pathogens likely depends on the specific AMF-pathogen interaction. Devi et al. (2022) found that combining AMF with endophytes reduced wilt incidence by 77%, yet such high efficacy may not be universal across pathosystems. These inconsistencies highlight the importance of optimizing AMF strains and application methods for tomato production in practice.

### 4.3 Biofertilizers

Biofertilizers are a category of fertilizers comprising microorganisms, substitute for conventional chemical fertilizers that enhance soil nutrients and facilitate nutrient absorption in crops (Kour et al., 2020; Mamouni et al., 2025). While the benefits of biofertilizers are well-documented, their widespread adoption faces several challenges that warrant critical examination.

Prolonged and excessive application of chemical fertilizers to mitigate the pest and disease effects, might result in environmental contamination and diminished food safety (Tallou et al., 2021; Jin et al., 2022). However, the claim that biofertilizers universally improve soil fertility and crop quality requires nuanced scrutiny. Although studies demonstrate that biofertilizers can enhance microbial activity, soil structure, and crop growth, their efficacy is highly dependent on environmental conditions, microbial strain specificity, and farming practices (Jin et al., 2022; Ruiz and Salas Sanjuan, 2022). For instance, the simultaneous use of biofertilizers with inorganic nitrogen fertilizers has been shown to improve tomato growth, with treated plants exhibiting significantly greater height, fresh weight, and dry weight compared to untreated controls. Yet, these results may not be replicable across all soil types or climatic conditions, raising questions about the generalizability of such findings.

Similarly, plant growth-promoting microorganisms (PGPM) and algal-based biostimulants markedly enhanced the soil fertility and yield of organic tomatoes. Specifically, PGPM-treated tomato plants showed enhanced characteristics including, height, leaf count, and root biomass, which attained 9.22 g per plant root biomass compared to 6.35 g per plant in the absence of PGPM application. The synergistic combination of PGPM with 1.0% algal biostimulant yielded 67.2 t/ha of tomatoes (Valentina et al., 2025). These outcomes may not account for variability in microbial survival rates in different soils or the potential for inconsistent product formulations in commercial biofertilizers. Additionally, tomato fruits treated with biofertilizers exhibited 40% higher soluble sugars, 23% increased vitamin C, and 62% reduced nitrate levels compared to those subjected to standard chemical fertilizers (Ye et al., 2020). However, long-term studies are needed to assess whether these benefits persist over multiple growing seasons or under stress conditions.

Biofertilizers generally enhance tomato growth and quality, but there can be adverse effects when they completely replace chemical fertilizers or in saline conditions. The detrimental consequences are primarily defined by imbalanced nutrient availability, soil microbial competition, and unfavorable impacts on various plant development metrics under saline stress (Deng et al., 2025; Pan et al., 2025; Tian et al., 2025). This implies that biofertilizers do not represent a universally applicable solution and may necessitate supplementary chemical inputs in specific agroecosystems to achieve optimal efficacy. Therefore, while biofertilizers offer a sustainable alternative to chemical fertilizers, their application must be carefully optimized, considering soil-specific conditions, microbial compatibility, and integrated nutrient management strategies. Overstating their benefits without addressing these limitations could lead to unrealistic expectations and suboptimal agricultural outcomes. Future research should focus on long-term field trials, standardization of biofertilizer formulations, and tailored recommendations for different cropping systems to ensure their effective and sustainable use.

## 5 Soil amendments in tomato cultivation

### 5.1 Biochar

**Figure 3:**
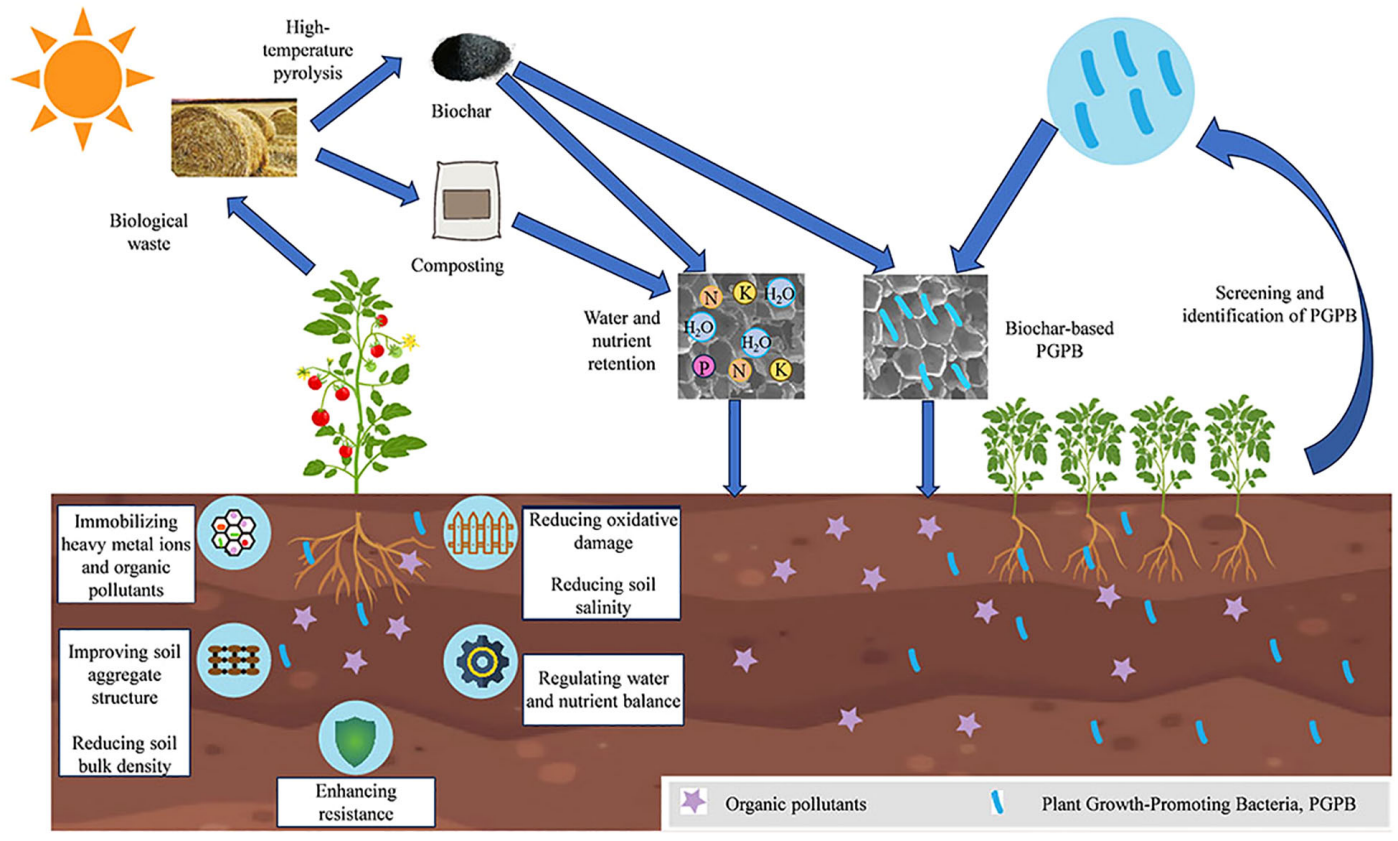
Schematic diagram of the role of soil amendments in remediating soil pollution and promoting tomato growth.

Biochar, a source of rich organic matter and minerals, significantly influences tomato growth and yield by enhancing soil structure and fertility (**Figure 3**) (Carril et al., 2025; Parasar and Agarwala, 2025; Waheed et al., 2025). It is the porous structure facilitates water and air retention in the soil, fostering an optimal growth condition for the tomato root system (Ikram et al., 2024; Waheed et al., 2025). However, the extent of these benefits may vary depending on soil type, biochar feedstock, and pyrolysis conditions, suggesting that universal applicability cannot be assumed.

Furthermore, biochar augments enzyme activity in the soil, which is crucial for the decomposition of organic matter and nutrient transformation. This increasing enzyme activity improves the soil’s detoxification capacity, which facilitates the removal of harmful substances, and foster healthier growth conditions for tomato plants (Pokharel et al., 2020; Murtaza et al., 2024). However, the long-term stability of these effects remains uncertain, as the impact of biochar on microbial activity may diminish over time, necessitating further research on its sustained benefits.

Biochar with particle sizes less than 3 mm enhanced 69% tomato fruit yield and improved key fruit quality parameters, particularly fruit diameter and carotenoid content by a remarkable 210% increase in soil organic matter, 100% mineral nitrogen content, available phosphorus by 29%, and available potassium by 30% (Zeeshan et al., 2020). While these results are impressive, it is important to consider whether such high gains are replicable across different agricultural systems or if they are context-specific. Furthermore, biochar was shown to effectively alleviate the concentrations of heavy metals such as copper, nickel, and cadmium in the soil, significantly reducing their levels compared to untreated soil. This alleviation significantly decreased the bioavailability of these heavy metals, thereby mitigating their toxic impact on tomato plants and promoting healthier growth (Alam et al., 2021; Zhou et al., 2022; Pir Dad et al., 2024). However, the mechanisms underlying this reduction, including potential roles of adsorption, pH modification, or microbial mediation, require further investigation to optimize biochar application in contaminated soils. Biochar application considerably enhanced tomato yield by up to 29.6%, increasing total soluble solids (TSS) and vitamin C content in the fruits (Lei et al., 2024). Various biochar types considerably influenced secondary metabolites which not only enhances tomato productivity but also elevates the nutritional quality of the tomato fruit (Petruccelli et al., 2015). Nevertheless, the variability in biochar feedstock (e.g., wood, crop residues, manure) and pyrolysis temperatures introduces complexity, as these factors significantly alter biochar’s chemical properties. For instance, biochar produced through pyrolysis at 550°C could enhance 42% fruit yield compared to the control group (Tartaglia et al., 2020). However, the optimality of this pyrolysis temperature across different biochar types has not been conclusively established. Biochar withnitrogen fertilizer enhanced the yield and quality of tomatoes while decreasing the quantity of nitrogen fertilizer utilized (Guo et al., 2021). This suggests potential economic and environmental benefits, but the optimal biochar-to-fertilizer ratio must be carefully calibrated to avoid unintended nutrient imbalances.

Conversely, biochar application positively affected tomato growth under saline stress conditions (**Figure 3**). Incorporating biochar effectively alleviated oxidative damage and enhanced the antioxidant capacity of plant, thereby enhance the growth and tomato yield (Kul et al., 2021; Yuan et al., 2023). Biochar application resulted in a 32% reduction in malondialdehyde levels and a 132% increase in peroxidase activity, indicating a substantial improvement in the plant’s antioxidant defense system under salt stress conditions (Coppa et al., 2024). Thus, while biochar shows the potential as a salinity mitigation tool, its efficacy in highly saline or arid regions warrants further validation.

### 5.2 Composting

Composting convert organic waste into stable organic additives appropriate for waste management at various scales (Sayara et al., 2020). While this process is widely promoted for its environmental benefits, its efficiency can vary significantly depending on feedstock composition, operational conditions, and microbial activity, which are often overlooked in generalized claims. The physicochemical qualities of compost and the succession of microbial communities can be markedly enhanced through the incorporation of mature compost (**Figure 3**) (Wang et al., 2022). However, the practicality of this approach may be limited by the availability of mature compost in resource-constrained settings, raising questions about scalability. Composting alleviates the environmental impact of agricultural waste and fosters agricultural sustainability by improving soil fertility and facilitating crop development. The significance of composting in the circular economy has been underscored by assessing its efficacy in managing organic waste and its leachate in practical scenarios (Oueld Lhaj et al., 2024). Nevertheless, the long-term effects of compost application, including potential heavy metal accumulation and nutrient runoff, are not always adequately addressed in existing studies. Similarly, although composting is framed as a strategic tool for sustainable agriculture (Bian et al., 2019; Sharma et al., 2024; Tahsini et al., 2024). Additionally, its economic feasibility for small-scale farmers remains debatable, particularly in the absence of composting production and distribution infrastructure.

The cultivation environment and soil quality significantly influence tomato growth, whereas compost is widely recognized as an excellent method to substantially improved many physiological markers and improve tomato yield (**Table 2**). However, the variability in compost quality (e.g., nutrient content, stability) complicates its standardized use. For example, vermicompost derived from cattle dung has been shown to improve soil structure and address agricultural challenges (Aksakal et al., 2016; Głąb et al., 2025). But its effectiveness depends on feedstock purity (e.g., antibiotic-free manure) and processing methods, which are not always guaranteed. Application of vermicompost significantly reduce soil bulk density and increase the content of water-stable macroaggregates, particularly in the 2.0–3.0 mm and 0.5–1.0 mm size fractions. Vermicompost can improve soil structure and porosity and enhance aggregate stability, which are key factors in improving soil quality and potentially increasing crop yields (Aksakal et al., 2016; Zhang et al., 2023). Nevertheless, the long-term sustainability of these benefits is uncertain, as repeated application may alter soil microbial communities in ways that are not yet fully understood. Combined application of 30% chemical fertilizer and 70% cow manure compost compared to chemical fertilizer significantly improved soil nutrientswith an elevation of 46%, 312%, and 46%; nitrogen,phosphorus, and organic matter, respectively. This treatment also enhanced tomato yield by 17% to 69% compared to pure chemical fertilizer (Aksakal et al., 2016; Hasnain et al., 2020; Zhang et al., 2023). This also demonstrates that compost application can be highly beneficial for tomato cultivation.

**Table 2:** The effects of different types of compost on tomato growth.

Compost types	Composting methods	Composting time	Application effects	References
Zizania latifolia leaf compost	Add enzymatic bacteria speed rotting agent	21 days	The optimal treatment of applying wild rice leaf compost increased soluble protein by 31.93%, Vc by 36.64%, soluble sugar by 18.55%, and sugar-acid ratio by 23.92% compared with commercial organic fertilizer.	(Chen et al., 2023)
Tomato straw compost	Addition of crude cellulose-degrading bacteria	60 days	The 3% compost treatment promoted tomato root development and seedling growth with the best results. 3% compost application significantly increased root length by 52.98%, root volume by 102.69%, and root surface diameter by 89.87%	(Yang D et al., 2024)
ermicomposting in situ	Add the earthworm species Akako Aiso Earthworms.	3 years	Soil total nitrogen increased by 125%, total phosphorus by 100%, total potassium by 57.14%, total carbon by 80%, adequate nitrogen by 160%, effective phosphorus by 240%, and fast-acting potassium by 600%	(Cao et al., 2022)
Tomato waste compost	–	90 days	1% tomato waste compost + chemical fertilizers increased yield by 28.9% over chemical fertilizers only	(Durmuş and Kızılkaya, 2022)
Water hyacinth and cow manure compost	Using drum composting	30 days	Tomato yield in the control group was 6.50 t/ha, drum composting 13.67 t/ha, an increase of about 110.6%	(Goswami et al., 2017)
Municipal organic waste	In-vessel decomposition with curing in windrows	10 weeks	Replacing mineral fertilizers with compost in greenhouse tomato cultivation maintains yield and quality, improves soil health, reduces water and pesticide use, and minimizes environmental impact by avoiding landfill waste.	(Martínez-Blanco et al., 2011)
Pig manure and corn straw compost	The addition of indole-3-acetic acid (IAA)-producing	41 days	Indole-3-acetic acid (IAA)-producing bacteria were obtained by screening for application in pig manure composting, and the screened IAA-producing bacteria had an enormous colonization potential in the composting process. The germination of tomato seeds and seedlings’ early growth and development were effectively assisted, and the compost quality was improved.	(Cai et al., 2022)

The compost industry is anticipated to undergo significant expansion and evolve towards greater specialization, scalability, and intelligence. Cocurrently, advancements in composting technology will prioritize high efficiency and environmental sustainability. This includes enhanced conversion efficiency, reduce greenhouse gas emissions and pollutant discharge, and improvements in the composition and biological activity of the resulting fertilizers.

### 5.3 Microbial agents

The application of microbial agents to improve tomato growth and yield has garnered significant attention as a novel research focus. In agricultural practices, bioactive compounds are critical components of tomato fruits, and microbial agents enhance soil quality through various mechanisms (Tóth et al., 2024). Microbial agents directly or indirectly improve soil microbiota, enhance nutrient availability, improve disease resistance, yield, and fruit quality in tomato plants (Meshram and Adhikari, 2024; Beyari, 2025). Microbial agents can promote tomato plant growth (Yan et al., 2024).

Cheraghi et al. (2023) demonstrated that in greenhouse experiments under high chemical fertilizer conditions, the combined application of vermicompost, PGPR and AMF significantly enhanced tomato root growth, zinc/iron uptake and soil respiration. This study systematically validated the synergistic mechanisms among organic inputs, microorganisms, root systems and plants at four interconnected levels. In addition, microbial agents have been used in tomato cultivation to control diseases (Tiwari et al., 2024; Zenelt and Krawczyk, 2025). Many microbial agents in tomatoes have exhibited considerable inhibition of wilt, green wilt, early blight, root-knot nematode, and bacterial wilt (**Table 3**). The primary parameters impacting microbial agents to enhance yields are complex and variable. Plant-growth-promoting inter-root bacteria (PGPB) produce various chemical compounds that diminish reliance on synthetic fertilizers and enhance tomato growth (Cochard et al., 2022). Beneficial soil fungi, specifically the fungal strains Trichoderma afroharzianum T22 and Funneliformis mosseae enhanced tomato yield by 13% and 15%, respectively (Minchev et al., 2024). These microorganisms are a viable sourceto diminish reliance on artificial fertilizers and pesticides by directly enhancing plant nutrient absorption and indirectly stimulating plant defense mechanisms. However, the field performance remains inconsistent due to variations in environmental conditions, soil microbiomes, and farming practices.

**Table 3:** Inhibition of tomato diseases by different microbial agents.

Microbial agents	Methods of application	Soil conditions	Types of disease	References
*Erythrobacter* sp. YH07	Cow dung compost with rice straw composting	Vegetable production greenhouse soil (containing pathogenic bacteria of tomato wilt)	Tomato fusarium wilt	(Tang et al., 2023)
*Bacillus siamensis* QN2MO-1	As a biological control agent alone	Tomato field soil was sieved and treated with three days of exposure to sunlight.	Tomato fusarium wilt	(Zhang et al., 2024)
Multiple functional strain combinations of *Bacillus*	Application of fungicide suspensions to tomato roots	Natural mountain black and red soils	Tomato bacterial wilt	(Guo et al., 2024)
*Bacillus velezensis* YXDHD1-7	Bacterial suspension is applied directly to tomato plants.	–	Tomato early blight	(Li et al., 2024)
*Trichoderma harzianum* agent and *Paecilomyces lilacinus* complex agent	Together with organic fertilizers (organic fertilizers are made from Hartz mycorrhizal fungicides mixed with well-rotted cow and sheep manure)	In greenhouses with high root-knot nematode disease	Tomato root-knot nematode disease	(Yang B et al., 2024)
*Aspergillus tubingensis* GX3	Seed coating is applied in a manner.	–	Tomato root-knot nematode disease	(Sikandar et al., 2023)
*Bacillus subtilis* (strain R31)	Injection of R31 fermentation broth into the inter-root soil of tomato plants	One is to use sterilized mixed nutrient soil (nutrient soil mixed with vermiculite in a 1:1 weight ratio). Another is to use yellow clay soil and vegetable planting soil (3:1 weight ratio mix)	Tomato bacterial wilt	(Sun et al., 2023)

Beyond biological limitations, economic and practical barriers hinder widespread adoption. Commercial microbial formulations often struggle with shelf life, precise application timing, and farmer accessibility compared to conventional agrochemicals. Furthermore, the regulatory for microbial inoculants remains underdeveloped in many regions, creating uncertainty for growers. To realize the full potential of microbial agents, future research should prioritize field validation under diverse conditions, optimize microbial consortia for stability and synergy, and develop cost-effective delivery systems that align with existing agricultural practices. Without addressing these gaps, microbial agents risk remaining a promising but underutilized tool in sustainable tomato production.

## 6 Conclusions

Effective nutrient management is critical for advancing sustainable tomato production, but future research better prioritize precision strategies tailored to varietal needs, growth stages, and environmental conditions. By bridging the gap between laboratory research and field application, this integrative approach has the potential to revolutionize tomato production systems, making them more adaptive to climate variability, resource constraints, and market demands. Key focus areas include optimizing dynamic nutrient formulations using real-time soil sensors and modeling to enhance uptake efficiency while minimizing waste. Additionally, organic fertilizers require standardization through improved composting techniques such as microbial consortia augmentation to ensure stability, safety, and consistent effects on yield and stress resistance. Field trials should validate these approaches under diverse agro-ecosystems fostering widespread adoption and enhancing sustainability of agricultural practices.

Biostimulants offer a promising pathway to reduce chemical dependency, but their mechanisms of action demand deeper investigation. Future studies should integrate multi-omics approaches including transcriptomics, metabolomics and microbiome analysis provides unprecedented insights into tomato physiological responses to nutrient management strategies. Metabolomic profiling for instance can reveal how specific nutrient formulations influence secondary metabolite synthesis and thereby link management practices to fruit quality attributes. Similarly microbiome sequencing elucidates how soil amendments modulate rhizosphere communities to enhance nutrient uptake and disease resistance. These methods deepen our understanding of plant–soil–microbe interactions while facilitating the development of precision nutrient management systems tailored to varietal needs and environmental conditions. Concurrently, research must explore the long-term impacts of organic amendments, such as biochar and cover crops on rhizosphere microbial communities using high-throughput sequencing. Understanding these interactions will enable microbiome engineering to enhance nutrient cycling and disease suppression while maintaining soil health.

To accelerate progress, interdisciplinary collaboration is essential, combining biotechnology, nanotechnology and data-driven tools for precision agriculture. Short-term efforts should focus on validating sensor-based nutrient models and biostimulant efficacy in controlled trials, while mid-term goals include piloting microbial-engineering approaches and nano-encapsulated nutrient delivery systems. Long-term strategies must integrate successful innovations into scalable farming practices and policy frameworks, ensuring global tomato production meets quality and sustainability targets. By adopting this structured yet adaptable roadmap, research can address current inconsistencies in yield and quality while promoting food security and ecological resilience.
